# Validating curricular competencies in innovation and entrepreneurship for biomedical research trainees: A modified Delphi approach

**DOI:** 10.1017/cts.2019.390

**Published:** 2019-07-29

**Authors:** Jane Garbutt, Alison Antes, Jessica Mozersky, James Pearson, Joseph Grailer, Emre Toker, James DuBois

**Affiliations:** 1 Department of Medicine, Washington University in St. Louis, St. Louis, MO, USA; 2 Department of Pediatrics, Washington University in St. Louis, St. Louis, MO, USA

**Keywords:** Delphi process, competencies, entrepreneurship, innovation, biomedical

## Abstract

**Introduction:**

Biomedical researchers need skills in innovation and entrepreneurship (I&E) to efficiently translate scientific discoveries into products and services to be used to improve health.

**Methods:**

In 2016, the European Union identified and published 15 entrepreneurial competencies (EntreComp) for the general population. To validate the appropriateness of these competencies for I&E training for biomedical researchers and to identify program content, we conducted six modified Delphi panels of 45 experts (6–9 per panel). Participating experts had diverse experience, representing such fields as entrepreneurship, academic research, venture capital, and industry.

**Results:**

The experts agreed that all 15 EntreComp competencies were important for biomedical research trainees and no additional competencies were identified. In a two-round Delphi process, the experts identified 120 topics to be included in a training curriculum. They rated the importance of each topic using a 5-point scale from not at all important (1) to extremely important (5) for two student groups: entrepreneurs (those interested in starting their own ventures) and intrapreneurs (those wanting to be innovative and strategic within academia or industry). Consensus (mean importance score >4) was reached that 85 (71%) topics were of high importance for the curriculum. Four topics were identified by multiple panels for both student groups: resiliency, goal setting, team management, and communication skills.

**Conclusions:**

I&E training for biomedical trainees should address all 15 EntreComp competencies, including “soft skills,” and be flexible to accommodate the needs of trainees on different career trajectories.

## Introduction

Today’s biomedical research trainees often need skills in innovation and entrepreneurship (I&E). Those who choose an academic career need I&E skills to equip them for success in an increasingly impact-orientated funding environment. Those who choose a career in industry or government need I&E skills to help them to identify, assess, and capitalize on opportunities to improve human health. To keep pace with the changing training needs to support multiple career options for biomedical researchers, the National Institutes of Health (NIH) has encouraged breadth and flexibility in research training programs [1]. We received a grant from the NIH to develop a competency-based program to introduce biomedical research trainees to innovation and entrepreneurial thinking. To achieve this goal, we first sought to identify the core I&E competencies a successful biomedical researcher should possess and to identify topics to be included in a curriculum to introduce these skills to biomedical research trainees.

In a preliminary review of the literature, we found that few publications addressed competencies for I&E; and we found none that specifically addressed the needs of biomedical researchers. Our study team considered the literature and their combined experience in I&E, to develop an initial working draft of I&E competencies shown in Table 1. After the grant was awarded, we updated our literature review and discovered the work of another group, “EntreComp: The Entrepreneurship Competence Framework” [27]. The EntreComp Framework is unique in the literature in that the aim was to identify competencies that would generate an “entrepreneurial mind-set” for all citizens rather than training entrepreneurs. Fifteen high-level competencies were identified using a rigorous mixed-methods approach, including a literature review and in-depth case study analyses leading to the development of draft competencies and a conceptual model, with validation via expert and stakeholder consultation [27]. Entrepreneurship is defined broadly as “the capacity to act upon opportunities and ideas to create value for others. The value that is created can be social, cultural, or economic” [28]. The authors of the EntreComp Framework suggest that it can be used as a “starting point for the interpretation of the entrepreneurship competence” and that it should be adapted and tailored to address the needs of specific groups. As of March 2018, the Framework had been used in at least 74 training programs [28]. The EntreComp competencies are provided in Table 2.

**Table 1.** Innovation and entrepreneurship (I&E) competencies for biomedical researchers: A working draft model

Type	Competencies
Cross-cutting personal competencies	Creativity and cognitive adaptability [2–5] Ethical problem-solving [6–8] Self-leadership and self-management [9–15] Social intelligence [16–18]
Team and project management competencies	Communication [3, 16] Leading creative and diverse teams [2, 19–21] Leading for innovation [2, 3, 22–25] Project management skills [3]
Business competencies	Business acumen and technical knowledge Negotiation Managing ethical and legal issues in business [26]

**Table 2.** Delphi panel domain areas and EntreComp competencies

Panel	EntreComp competencies	Definitions^a^
1. Management (*N* = 8)	Planning and management	Set goals and define priorities and action plans, while adapting to unforeseen changes
	Financial and economic literacy	Estimate the cost of bringing an idea to fruition, and make financial plans and decisions that ensure the activities can continue over the long term
	Mobilizing resources	Obtain and manage the resources needed to turn ideas into action, and make the most out of limited resources
2. Vision and imagination (*N* = 9)	Spotting opportunities	Identify opportunities to address problems, challenges, and needs, and judge whether to act on them
	Vision	Imagine the future and visualize strategies to turn ideas into action
	Valuing Ideas	Recognize an idea’s potential to create value, and identify suitable ways to share and protect that idea
	Creativity	Develop multiple ideas and experiment with innovative approaches to solve existing and new challenges
3. Social skills (*N* = 7)	Self-awareness and self-efficacy	Reflect on personal strengths, weaknesses, needs, and aspirations, and believe in the ability to influence course of events
	Mobilizing others	Inspire relevant stakeholders through effective communication, persuasion, negotiation, and leadership to obtain the support needed to achieve outcomes
	Working with others	Network and cooperate with others to develop ideas and turn them into action
4. Psychological skills (*N* = 7)	Learning through experience	Reflect on experiences and learn from successes and failures
	Taking the initiative	Initiate activities and take on challenges to achieve goals
	Motivation and perseverance	Be determined and focused to turn ideas into action and reach long-term goals, even under adversity and failure
5. Ethical and decision-making skills (*N* = 8)	Coping with uncertainty, ambiguity, and risk	Make decisions despite incomplete information and unknown results
	Ethical and sustainable thinking	Assess the consequences of ideas and activities on communities, markets, society, and the environment, and reflect on the sustainability of social, cultural, and economic goals
6. EntreComp competencies (*N* = 7)	All competencies	

a Definitions are those presented to the expert panels and are adapted from the EntreComp Framework [27].

We decided to use the EntreComp Framework as a starting point to develop our I&E training program for biomedical research trainees for the following reasons: (1) the aim of the EntreComp Framework to generate an entrepreneurial mind-set was in line with our intent; and (2) all the competencies in our working draft were contained within the Framework. To validate the appropriateness of the EntreComp competencies for I&E training for biomedical research trainees in the USA and to identify course content for our program, we conducted six modified Delphi panels. Delphi panels are typically used to establish group consensus about priorities when many options exist, and modified Delphi processes have previously been used to develop curricula [29, 30–35], which was our purpose. We needed expert opinions upon which to build our curriculum.

## Materials and Methods

### Compliance

The project was presented to the Washington University in St. Louis (WU) Institutional Review Board who determined that it did not constitute research because it aimed at producing consensus among experts rather than generalizable knowledge from subjects. However, all participants freely agreed to participate in the process and provided permission to publish their names and biographical details, which they reviewed and approved (see Appendix A).

### Participants

Panelists were selected using purposeful, non-probability sampling with the goal of recruiting a heterogeneous group of experts from the USA. Forty-five experts were identified (from academia, 25; venture capital, 11; industry, 9). Many had experience in biomedical entrepreneurship as shown in their biosketches (see Appendix A). All experts agreed to participate in a modified Delphi process over 2 months, with a total time commitment of less than 2 hours. A $100 Amazon gift card was offered as an honorarium.

### Procedures

Recognizing the breadth of the I&E competencies, and to avoid overburdening panel members, we grouped the EntreComp Framework high-level competencies into five domain areas (Table 2). Six Delphi panels were planned, one for each domain area (Panels 1–5) and one to validate the 15 EntreComp competencies for use in our program (Panel 6).

The project team allocated the 45 experts to the six panels based on their area of expertise and entrepreneurial experience to ensure that each panel had a mix of both content experts and experienced entrepreneurs. Panel size ranged from 6 to 9 and is provided in Table 2. Between April 9, 2018 and July 18, 2018, each panel worked independently and simultaneously with panelists blinded to the identity of other participants. For each round of the Delphi process, individual panelists accessed the surveys hosted in Qualtrics through unique links. Each round allowed a 2-week window to submit responses with reminder e-mails and a 1-month gap between rounds.

For all panels, at the beginning of round 1, all experts were asked to review background information to understand the context and purpose of the Delphi panel project. First, they were introduced to the program goal: to better equip biomedical research trainees for their future careers by teaching them I&E skills. Second, they were introduced to the EntreComp Framework and the 15 high-level competencies that we had grouped into five overarching categories: management, vision and imagination, social skills, psychological skills, and ethical and decision-making skills. Finally, the experts were asked to make the following assumptions: “(1) All course participants are enrolled in a training program to pursue a career in biomedical research. (2) They may work in the university, government, industry, and other settings. (3) They may or may not become entrepreneurs. (4) The course they were considering would be introductory, and approximately 20 hours long.”

### The EntreComp Framework Panel

The goal of this panel was to validate the EntreComp Framework as a whole for biomedical researchers. The seven panelists were provided a list of all 15 EntreComp Framework high-level competencies and a brief description of each (Table 2). They were asked whether each competency was relevant for biomedical research trainees (yes/no); in the case of a “no” vote, they were asked to provide a rationale. Panelists were also asked if any additional competencies should be added to the list. A priori, we defined agreement among the experts that the EntreComp competency was relevant to biomedical research trainees as a yes vote from 5/7 panel members. This approach is consistent with other Delphi methodology [36]. We chose to use this variation on the Rand criteria due to our sample size and dichotomous choice. If new competencies were identified by individuals, they would be presented to the panel in a second round to evaluate for consensus.

### Panels 1–5

The goal for Panels 1–5 was to identify topics to be included within the training program for the EntreComp competencies assigned to that panel. In round 1, participants were presented with their panel’s EntreComp high-level competencies and brief definitions (Table 2). Then they were asked “What content do you think should be taught? Content can be described as topics, knowledge, skills, or attitudes. Please list as many content areas that you think should be included.”

For each panel, responses from round 1 were analyzed to eliminate redundancy and the responses were summarized. In round 2, panelists were asked to review the summarized list and rate the importance of each topic for an introductory course in entrepreneurial thinking for biomedical research trainees using a 5-point scale from not at all important (1) to extremely important (5). For each topic they provided their rating for two student groups: entrepreneurs (those with an interest in starting their own ventures) and intrapreneurs (those who want to be innovative and strategic within pre-established companies or an academic career) [37]. For each panel, the mean importance score of all expert panelists was calculated for each topic. We defined topics with a mean importance score > 4.0 (very or extremely important) as having a consensus that the topic was “highly important” to teach. This approach is consistent with other Delphi methodology that defines a consensus using a mean score [36]. We adopted a 5-point rating system (rather than 9 point) to make it easier to label each option. Participants were also asked to use open-ended textboxes to provide other content areas they thought were missing from the list.

After analyzing results from round two, it was decided that a third round for Panels 1–5 was not required as the results were intended to guide curriculum development by providing an expert generated list of topics, rather than build consensus.

## Results

### Validity of EntreComp Competencies for Biomedical Research Trainees

The seven experts on the EntreComp Framework panel agreed that the 15 EntreComp competencies were all important for biomedical trainees. After analyzing results from round 1, we determined that a second round for this validation panel was not required as no new high-level competencies were identified.

### Panels 1–5

Altogether 207 topics were generated across the five panels in round 1, resulting in 120 summarized topics (17–31 per panel) for analysis in round 2. Overall, 36/38 (95%) experts submitted responses for both rounds of the Delphi process.

### Course Content

The five panels achieved consensus that 85 (71%) of the 120 topics ranked in round 2 were of high importance to include in the curriculum. These included 42 (49%) topics considered to be of high importance for all biomedical research trainees regardless of future career direction, 35 (41%) additional topics for entrepreneurs only, and 8 (9%) topics for intrapreneurs only (Table 3). In Table 4, topics that reached consensus for entrepreneurs and intrapreneurs are listed by panel grouped by importance for trainee’s career direction (both entrepreneur and intrapreneurs, entrepreneur only, and intrapreneur only). The complete data for all panels are provided in Appendix B.

**Table 3.** Number of topics assessed in round 2 of the modified Delphi process by panel

Panel	Total topics scored	Number of topics that reached consensus			
		Overall	Both E&I	E-only	I-only
Management	23	15	5	10	0
Vision	17	14	6	8	0
Social skills	27	20	11	7	2
Psychological skills	22	17	12	2	3
Ethical and decision-making skills	31	19	9	7	3
Total	120	85	42	35	8

E, entrepreneurship; I , intrapreneurship.

**Table 4.** Innovation and entrepreneurship (I&E) topics that reached consensus, by panel

	Panel 1	Panel 2	Panel 3	Panel 4	Panel 5
	Management	Vision and imagination	Social skills	Psychological skills	Ethical and decision-making skills
Consensus for both entre- and intra-preneurs	Team management skills. Goal setting. Decision making skills. Self-management skills. Project management skills	Communication skills to illicit different perspectives. Identify assumptions of business plan. Problem formulation strategies. Know the state of your industry. How to screen ideas. How to think divergently and come up with many solutions	Formal presentation skills. Active listening skills. Give and receive feedback. Conflict resolution. Goal setting. Team dynamics. Improvisational dialogue skills. Courtesy skills. How to develop meaningful interpersonal relationships. How to be assertive. Awareness of your emotions and ability to control your reactions to those emotions	Make the most of your strengths and capabilities. How to deal with failure. Methods to measure progress to inform future decisions. Self-management skills. Networking skills. Use goal setting as motivational strategy. Grit and perseverance. Be aware of cognitive biases. Develop team trust. Experimental skills to test hypotheses. How to anticipate problems. How to coach others	How to recognize and take stock of your circumstances. How to ethically interact with patients. Ethical interactions with industry and institutions. Ethical pragmatism. Intellectual property issues. Questioning your own and other’s judgment: understanding biases. Know unmet health needs. Develop an experimental mind-set. Know how to interact ethically with other professionals
Consensus for entrepreneurs only	Knowledge of the different types of funding sources, and how and when to apply for them. Team building. How to define the resources you need for your venture. Facilitation skills. Fundamentals of product adoption. Fundamentals of intellectual property. Fundamentals of product approval. How to pitch an idea or otherwise build, improve, and defend business cases. How to create a business plan. How to work with outside partners and institutions	How to identify your minimum viable product (MVP). How to perform market research. Understand what inspires and motivates you to act. Interview techniques for different audiences. Know what investors want to see before investing. Know market trends and underlying models of supply and demand. Know entrepreneurial ventures in your field. How to find uses for existing technologies	Networking skills. Survival skills such as resilience, managing change, and risk management. Negotiating skills. How to build a personal brand. How to utilize body language effectively. How to create and deliver elevator pitches. Establish norms and expectation of the team	Self-efficacy. Person-environment fit	Resiliency skills. Seek outside help to make decisions. Establish your central vision and refer back to it to guide decision making. Anticipate worst case and most likely consequences of your actions. Implementation considerations, being thrifty when expanding in new directions. Stay up to date with global issues. Know a framework for rational economic decision making, such as Net Present Value
Consensus for intrapreneurs only			How to lead when you don’t have a position of authority. How to “manage up”	Give/receive feedback. Sense-making within teams. Emotional intelligence: manage your own and others emotions	Conflicts of interest and ethical issues underlying them. Moral reasoning. Cross-cultural communication

Although each panel was assigned different EntreComp competencies in rounds 1 and 2, several topics were identified as high importance to include in the curriculum for both career groups by multiple panels. For entrepreneurs, these included resiliency/self-management (four panels), goal setting (three panels), team management (three panels), and communication skills (three panels). These same topics were identified by multiple panels for intrapreneurs, resiliency/self-management (two panels), goal setting (three panels), team management (three panels), and communication skills (four panels).

## Discussion

The six modified Delphi panels reached our two aims – to identify the I&E competencies a biomedical researcher should possess and to identify topics to be included in the core curriculum to introduce I&E skills to biomedical research trainees. Our findings suggest our introductory training program should address all 15 EntreComp competencies, yet be flexible to accommodate variation in needs of trainees on different career trajectories. One approach to meet the need for breadth and depth of course offerings is to provide both core and elective courses.

Our expert panelists provided guidance about topics to be included in core and elective courses. Half of the 85 topics identified as high importance were ranked as high importance for all trainees, regardless of their career trajectory. Reviewing this topic list suggests that core courses should be designed to teach trainees the following I&E skills: how to identify opportunities for innovation; how to determine their potential for success; how to communicate about your innovation idea to various audiences; how to build and manage teams; how to be aware of the ethical consequences of your decisions and actions; and self-management and resiliency. Topics identified as high importance for only one career group could be considered as electives, allowing trainees to tailor their program to meet their needs. Skills of particular interest to entrepreneurs might be learned through elective courses focused on commercialization such as identifying your funding needs and opportunities, and building a business plan. Elective courses of interest to intrapreneurs might include managing up and social entrepreneurship including cultural competencies. Elective courses also provide opportunity for more in-depth coverage of topics that might be relevant only for some trainees.

With the goal of improving human health through translational innovation, I&E skills are important for the translational research workforce of the future [38]. An important initiative in this regard is the Clinical and Translational Science Award (CTSA) program with the goal of efficiently translating research knowledge into improved health [39]. Several core I&E skills identified by our experts have previously been identified as core competencies for clinical and translational science, including the “soft skills” needed to function as a professional and to work in multi- and interdisciplinary teams [38, 40–42]. Courses in communication and team science are provided at many CTSA sites across the country as part of degree programs in clinical and translational research [38, 40, 41]. Training in the more traditional entrepreneurial skills such as design thinking and commercialization is offered through engineering and business schools [43]. For some there is a tension between the goals of medicine and science and entrepreneurship. Medicine and science are founded on goals such as improving health, creating generalizable knowledge, openness, and transparency [44–46]. These goals may conflict with goals of entrepreneurship where profits and financial motives may lead to secrecy, proprietary claims, and competition rather than collaboration [28, 29, 47]. Evidence has shown that financial incentives and motives can affect decision making, change behaviors, and potentially lead to unethical actions, for instance through conflicts of interests [29, 46, 48, 49]. At the same time, collaborations between industry and academia are now the norm, alongside an emphasis on translational science, suggesting we should not abandon these relationships but rather we need to ensure that individuals are aware of these tensions [47, 49, 50]. This is our rationale for including ethics experts in our panels and for requiring an ethics course for all students, regardless of track. Our web-based introductory program will increase opportunity for pre-and post-doctoral students to access training in I&E skills that are relevant for translational researchers. The final program will include a bootcamp and a team-based capstone project to provide learners opportunities to integrate their new knowledge and skills.

It is notable that the topics of self-management and resilience were rated as highly important for both entrepreneurs and intrapreneurs across multiple panels. Our experts recognized resilience as an important skill to deal with the ups and downs of innovation. Indeed, entrepreneurial resilience has been shown to have a significant positive relationship with success for individuals and businesses [51, 52]. Overall, research suggests that resilience is a modifiable construct and not an inherent, immovable trait [53]. Encouraged by a recent metanalysis that found “a modest but consistent benefit of resiliency training programs in improving a number of mental health outcomes within three months of follow-up” [53], we plan to have a core course to improve resilience skills. We will carefully evaluate the courses as we note that the authors commented that the 13 randomized controlled trials included in the meta-analysis were small and generally of poor methodological quality [53].

## Limitations

Our panelists each had I&E experience relevant to biomedical research, but it cannot be assumed that a different panel of experts would reach the same conclusions regarding competencies and program content for an I&E curriculum for biomedical research trainees. We based the definition of highly important topics for learner groups on common sense and common practice [36], and changing this definition might change the conclusions drawn from the study. We provide the complete study data in Appendix B to allow the reader to review all topics suggested by the expert panelists, not just the ones that we identified as being highly important. We focused on identifying topics to include in our curriculum and did not ascertain the level of mastery expected of learners. Our program will be an introductory course for pre- and postgraduate students, and the learning objectives will determine the level of mastery expected.

## Conclusion

The six modified Delphi panels identified topics to be included in a basic training program to encourage an entrepreneurial mind-set in biomedical research trainees. We will use these findings to inform the development of our introductory program in I&E training targeting this group, specifically to inform learning objectives, course content, and designation as a core or elective course. While these training recommendations are based on the expert consensus, we will need to assess learner outcomes and reactions to evaluate the success of our program. Additional considerations such as how the course should be implemented (in-person, web-based, team-based), course duration, and the roll of additional components such as mentoring and a capstone project to synthesize learning need further exploration.

## Appendix A

### Panel 1: Management

Michael Bishop, PhD

michael.j.bishop@gsk.com

Dr. Bishop is the Director of Medicinal Chemistry at GlaxoSmithKline (GSK), where he works as part of the Discovery Partnerships with the Academia team: partnering leading academic researchers with experienced drug discovery scientists. This builds upon his 20+ years of experience in drug discovery research at GSK. Dr. Bishop earned his PhD in Chemistry at Rice University.

Brian Cudney, MBA, CSSBB

brian.cudney@cardinalhealth.com

Mr. Cudney is currently the Director of Chemistry, Manufacturing and Controls for Cardinal Health Regulatory Sciences. He has over 20 years of experience in the pharmaceutical industry and has worked at companies such as KV Pharmaceutical Company and Nesher Pharmaceuticals LLC. As part of his duties, he has managed and mentored scientists in industry settings. He earned his MBA with an emphasis in Marketing and Technology at the University of Connecticut.

Jeff Hersh, MD, PhD

jeff.hersh@ge.com

Dr. Hersh is currently the Chief Medical Officer of GE HealthCare. He has been board certified in Internal Medicine, Pediatrics, Emergency Medicine and Disaster Medicine and has over 30 years of clinical experience as a practicing physician. Previously, he has held faculty appointments at universities of Yale, Dartmouth, Cornell, Tufts, Massachusetts, and Harvard; he also has extensive experience in the medical device industry. He earned his MD at the Miller School of Medicine, University of Miami; and his PhD in Theoretical Physics at Yale University.

Jay Baumohl, MBA

jrbaumo@yahoo.com

Mr. Baumohl is the principal at Olive Street Advisers and the director of the St. Louis Arch Angels, the St. Louis region’s largest Angel investment group. Prior to his current position, he has held roles such as the Chief Financial Officer at Pharmacy Services, Inc. and the Vice President of Finance at Walgreens. All totaled, he has over 20 years of financial leadership experience. He earned his MBA in Finance at the Darden School of Business, University of Virginia, and a BSBA in Accounting at Washington University.

Michael Myers, PhD

myers_michael_r@lilly.com

Dr. Myers is the Senior Director in External Innovation at Eli Lilly, responsible for scientific due diligence, and he remains involved in academic outreach. He has over 30 years of experience in the pharmaceutical industry and has contributed as an inventor to over 35 patents. Dr. Myers has led drug discovery teams in the research of treatments for cancer, CNS, infectious, and cardiovascular diseases and is experienced in project and portfolio management. He earned his PhD in Organic Chemistry at the University of Pittsburgh.

Tomas Isakowitz, PhD

tomas@upenn.edu

Dr. Isakowitz is an Adjunct Assistant Professor of Computer Science at the Department of Psychiatry, Perelman School of Medicine, University of Pennsylvania. He is also the Founder and Manager of the Penn Center for Innovation Fellows Program and the Principal Investigator of the Penn I-Corps Site. He has taught “Building an Engineering Sciences Startup” and “Translational Therapeutics” and has extensive experience mentoring early entrepreneurial teams through the I-Corps program. He earned his PhD in Computer Science at the University of Pennsylvania.

Michael Poisel, MBA, MSSE

mpoisel@upenn.edu

Mr. Poisel is the Director of Penn Center for Innovation Ventures, where he helps to commercialize Penn technology and assists UPenn faculty and staff to launch and manage entrepreneurial ventures. Prior to this, Mr. Poisel made investments for NewSpring Capital, Apax Partners, and GE Capital. He earned his MBA in finance and entrepreneurial management at theWharton School of Business, University of Pennsylvania, and his Masters of Science in Systems Engineering from theMoore School of Engineering, University of Pennsylvania. He earned his BS in Mechanical Engineering from the Rose-Hulman Institute of Technology.

Tara Butler, MD, MBA

tara.butler@ascension.org

Dr. Butler is currently the Managing Director at Ascension Ventures, a strategic healthcare venture fund partnered with 474 acute care hospitals and other healthcare-related facilities in 40 states and the District of Columbia. Prior to her current position, she worked in business development at Medtronic and finance at Honeywell. Dr. Butler completed a residency in obstetrics and gynecology at Washington University, St. Louis. She earned her MD from the School of Medicine, University of Pennsylvania, and her MBA from theWharton School of Business, University of Pennsylvania.

### Panel 2: Vision and Imagination

Henry Chi, JD

henry.chi@capitalinnovators.com

Mr. Chi is currently the Portfolio Manager at Capital Innovators, a startup accelerator based in St. Louis. There, he manages a portfolio of over 90 companies. He earned his JD at St. Louis School of Law, Washington University.

Jordan Zipkin, BS, BA

jordan@realventures.com

Mr. Zipkin is currently the Operations Manager at Real Ventures, an early-stage venture capital firm located in Montreal, Canada. Prior to this, he was the Head of Business Intelligence at ML Partners, an investment group, and the General Manger of Sage, a healthcare supplies firm. He earned his bachelor’s degree from Washington University, St. Louis, with majors in Mathematics, Economics and Strategy, Operations and Supply Chain Management, Entrepreneurship, International Business, and Human Resource Management.

Jeffrey Gentsch, MBA

jeff@gentschcapital.com

Mr. Gentsch is currently the Managing Director of Gentsch Capital Partners (GCP), a lower middle market private equity firm based in St. Louis as well as a Venture Partner at Advantage Capital Partners. He has over 25 years of experience in private equity investing; and prior to founding GCP, he was a partner at Key Principal Partners, a middle market venture and growth capital equity firm, and the Managing Director of the Harbour Group, a middle market buyout firm. He earned his MBA at Olin Business School, Washington University, St. Louis.

Basil Horner, MBA

base.horner@archpartnersllc.com

Mr. Horner is currently a Managing Partner at Arch Partners LLC, an investment firm located in the Southwest of the US, and also heads the Screening Panel for Desert Angels, a Tucson, AZ-based angel investment firm. He has over 30 years of experience as an investment banker and angel investor and has successfully completed more than $6 billion in financing and advisory transactions for >100 middle-market companies. He earned his MBA with honors in Finance and Marketing from the Booth School of Business, University of Chicago.

Jackson Nickerson, PhD, MBA, MSME

nickerson@wustl.edu

Dr. Nickerson is the Frahm Family Professor of Organization and Strategy at the Olin Business School, Washington University, St. Louis, as well as a Nonresident Senior Fellow in Governance Studies at the Brookings Institution. He is the co-creator of Critical Thinking@Olin and teaches courses on innovation, organizational strategy, and critical and strategic thinking. Dr. Nickerson’s research focuses on organizational structure choices and performance; he has published over 30 peer-reviewed articles and has been the author or editor of five books. He has received numerous awards for both his teaching and research. He earned his PhD in Business and Public Policy and MBA in Strategy and Finance at the Haas School of Business, University of California, Berkeley, as well as an MSME from the College of Engineering, University of California, Berkeley.

Vijay Ramani, PhD

ramani@wustl.edu

Dr. Ramani holds several positions at Washington University, St. Louis. He is the Roma B. And Raymond H. Wittcoff Distinguished University Professor, the Director of the Center for Solar Energy and Energy Storage, and the Faculty Fellow for Entrepreneurship for the Danforth Campus. Dr. Ramani’s research interests focus on electrochemical energy conversion and storage, for which he has won many awards. He has over 100 publications in refereed journals and has taught classes ranging from chemical reaction engineering to renewable energy technologies. He earned his PhD in chemical engineering at the University of Connecticut.

Jennifer Silva, MD

jennifersilva@wustl.edu

Dr. Silva is currently an Associate Professor of Pediatrics, Cardiology and the Faculty Fellow in Entrepreneurship at School of Medicine, Washington University, St. Louis. She has published over 30 articles in peer-reviewed journals, and is the co-founder and CMO of SentiAR, a digital health, software device company and recipient of an NIH-SBIR Fast Track Grant. She earned her medical degree from School of Medicine, St. George’s University, completed her residency in pediatrics at Miami Children’s Hospital, Fellowship in Pediatric Cardiology at School of Medicine, Washington University, St. Louis, and a 4-year Advanced Fellowship in Pediatric and Adult Congenital Electrophysiology at Children’s Hospital Boston/Harvard Medical School.

Bijal Desai-Ramirez, MBA

bijal@wepowerstl.org

Ms. Desai-Ramirez is the Vice President of Entrepreneurship and Investments at WEPOWER, which catalyzes communities to build and leverage power to design education, economic, health, and justice systems that are just and equitable for all. Her role focuses on how we can all better engage, support, and accelerate the ideas of underrepresented entrepreneurs. She is also the Co-Founder of Filament LLC, a meeting facilitation and design company in St. Louis, MO. Prior experiences include serving as COO of Filament, the Founding Executive Director of Education Innovation at the University of Missouri-St. Louis, where she fostered the growing educational innovation and startup ecosystem, and work with Boys & Girls Clubs of Greater St. Louis, IRI/PepsiCo, Pfizer Inc., and as a startup, nonprofit, and corporate consultant. She earned her MBA with an emphasis in Strategy and Social Entrepreneurship from Olin Business School, Washington University, St. Louis.

Warren Seering, PhD

Seering@MIT.edu

Dr. Seering is the Weber-Shaughness Professor of Mechanical Engineering. He has helped to establish the MIT Machine Dynamics Laboratory, was a member of the MIT Artificial Intelligence Laboratory, and cofounded and for a time directed the Center for Innovation in Product Development at MIT. His research has spanned machine dynamics, engineering system design, and product development. Dr. Seering has won several awards for his teaching excellence, and he has mentored over 150 advanced degree-seeking students and taught courses on design, product development, applied mechanics, system dynamics, instrumentation, and computer software. He earned his PhD in Mechanical Engineering from Stanford University.

### Panel 3: Social Skills

Tamara Friedrich, PhD

Tamara.Friedrich@wbs.ac.uk

Dr. Friedrich is an Associate Professor of Entrepreneurship and Innovation at the University of Warwick. She teaches a core module in the Warwick Business School MBA program on Innovation and Creativity in Organizations, and also teaches Problem-Solving in Organizations to undergraduate students. She is also the Course Director for the Warwick Business School Foundation Year Program, which fosters equal opportunities for promising students from traditionally underrepresented backgrounds. Dr. Friedrich is an industrial and organizational psychologist, pursuing research centered on creativity and innovation in individuals and teams, as well as the role that leadership plays in these topics. Prior to her current responsibilities, she was an Assistant Professor and the Founder and Director of the Center for the Advancement of Creativity and Entrepreneurship, Savannah State University. She earned her PhD in Industrial and Organizational Psychology at the University of Oklahoma.

Joseph Steensma, MPH, EdD

jsteensma@wustl.edu

Dr. Steensma is a Professor at Brown School, Washington University, St. Louis, a Visiting Professor at the University of Wollongong, and a Senior Scholar at the Global Good Fund. He teaches classes in biostatistics, environmental health, and the public health implications of climate change. His research focuses on the intersection between economics, health, and environmental degradation. Prior to his academic career, Dr. Steensma founded and was the CEO for the Industrial Solutions Group until it was sold to Concentra in 2007. He has helped dozens of start-up companies, from all over the world, launch and grow with the purpose of improving the human condition and/or helping us live more sustainably. He earned his EdD focusing on Business and Leadership at Indian Wesleyan University and a MPH focusing on Biostatistics and Environmental Health at Saint Louis University.

Matt Homann, JD

Matt@TheFilament.com

Mr. Homann is the Founder and the CEO of Filament, a meeting facilitation and design company in St. Louis, MO, as well as the founder and advisor of Invisible Girlfriend and Invisible Boyfriend, a virtual companionship company. Prior to his current roles, he founded LexThink LLC, acted as the CEO for Kendeo, was a practicing lawyer and also taught at the School of Law, Washington University. He earned his JD at St. Louis School of Law, Washington University.

Ken Janoski, MBA

Ken@GuidanceVC.com

Mr. Janoski is the Managing Partner at Guidance Ventures, working with university researchers to commercialize discoveries, a SBIR/STTR Phase II Review Panelist for Business Commercialization at the National Science Foundation, and an Oversight Board Member for the Emory-Georgia Tech Coulter Translational Partnership. Over his career, he has co-founded or has been a venture investor in 12 bioscience companies, and held senior executive roles such as President, CEO, and Board Chairman as well as served as the President and CEO of BioGenerator, a philanthropic venture fund. Mr. Janoski earned his MBA with an emphasis in finance at Washington University, St. Louis.

Nichole Mercier, PhD

nmercier@wustl.edu

Dr. Mercier is the Managing Director of Office of Technology Management (OTM) at Washington University, St. Louis. She has worked for the OTM in many capacities, ranging from license and business development to managing external partnerships with the university to developing educational programs; she has also been a leader in the Women in Innovation and Technology Program. Dr. Mercier earned her PhD in Biomedical Sciences at the University of Massachusetts Medical School.

Carla Krause

carla.krause@cardinalhealth.com

Carla Krause is the Director at Cardinal Health, Grand Rapids, Michigan. Carla Krause has more than 15 years of medical device regulatory affairs and quality assurance experience. Previously, she worked as the Director of QA/RA at Vention Medical, Inc., and the VP of Regulatory Affairs and Quality Assurance at Aspen Surgical Products. Her experience includes executive management responsibility for global medical device manufacturing and contract manufacturing companies. She had responsibility for quality and regulatory due diligence and integration, along with collaboration and planning with all functional areas, for several successful acquisitions during her career. Her responsibilities included design control, document control, change control, risk management, inspection, manufacturing, assembly, packaging, labeling, sterilization, biocompatibility, environmental management, validation/verification, distribution, marketing, post-market surveillance activities, internal audits, customer audits, supplier audits, regulatory audits, supplier management, global submissions, licensing, registrations, and certifications for Classes I, II, and III medical devices including implantable devices. She has also worked in the clinical laboratory arena and IVD in the areas of Chemistry, Hematology, Microbiology, Virology, Mycology, Immunology, and Blood Banking. Her current areas of responsibility include CMC plant transfer activities, post-market variations, document control, special project group, and a full service of medical device service offering.

### Panel 4: Psychological Skills

Tammy English, PhD

tenglish@wustl.edu

Dr. English is currently an Assistant Professor at the Department of Psychological and Brain Sciences, Washington University, St. Louis. Her research focuses on understanding emotion regulation and relationships, and she has over 30 publications in refereed journals. She has won awards for her mentorship and has taught courses such as Emotion Regulation and Introductory Psychological Statistics. Dr. English earned her PhD in Social and Personality Psychology at the University of California, Berkeley, and she completed a post-doctoral fellowship at Stanford University.

Benjamin Baran, PhD

ben@benbaran.com

Dr. Ben Baran is an Assistant Professor of Management at Cleveland State University, a Co-Founder and the Principal of the consulting firm Indigo Anchor, and a Commander in the U.S. Navy Reserve. His research focuses on the intersection of human resource management and leadership and organizational change, and his consulting spans a wide range of sectors and industries. Dr. Baran earned his PhD in organizational science from The University of North Carolina at Charlotte.

Mary Jo Gorman, MD, MBA

mjgorman@prosperstl.com

Dr. Gorman serves on the board of Check-Cap (NASDAQ: CHEK) and TripleCare, the latter for which she also acts as the Interim-CEO. She is also the Lead Managing Partner of Prosper Women Entrepreneurs Startup Accelerator, an early-stage investor for women-led companies. In total, she has started four companies since 1991 and has over 20 years of clinical, healthcare management, and entrepreneurial experience. She completed her training in internal medicine at Southern Illinois University Affiliated Hospitals, and was board certified in internal medicine and critical care medicine. Dr. Gorman earned her MD from the School of Medicine, Southern Illinois University, and her MBA from the Olin School of Business, Washington University.

Kishore Kanakamedala, MBA, MS

kishore.kanakamedala5@gmail.com

Mr. Kanakamedala is the director of Product Management at Google. He has extensive leadership experience in programming, mergers and acquisition, and product management and has worked at companies such as McKinsey, SAP, Microsoft, and Aspen Technology. He earned an MS from Purdue University and an MBA from the Wharton School of Business, University of Pennsylvania.

Scott Bernstein, MBA

scott@lacventures.com

Mr. Bernstein is the Principal at Lewis and Clark Ventures, a venture capital fund serving the software, healthcare, and agriculture tech industries. He is a Kauffman Fellow of Class 22 and serves on the boards of OneSpace and Adarza BioSystems, Inc. Immediately prior to his current role as a principal, he was the Director of Operations at Capital Innovators, which built upon his diverse experience in product strategy and development, portfolio management, and private wealth management. He is active in the St Louis start-up ecosystem, serving on the Arch Grants Competition Committee and as a judge for Washington University startup competitions. Mr. Bernstein earned his MBA at the Booth School of Business, University of Chicago.

David Roth, MD, PhD

david.roth2@uphs.upenn.edu

Dr. Roth is the Simon Flexner Professor of Pathology, the Chair of the Department of Pathology and Laboratory Medicine, and the Director of the Penn Center for Precision Medicine at the Perelman School of Medicine, University of Pennsylvania. He has been awarded several honors for his excellence in teaching and runs a robust research laboratory. His research and clinical interests include DNA repair, cancer genetics, and gene rearrangements during lymphocyte differentiation. He has published over 100 articles in peer-reviewed journals and served as an editor of Immunology, 7^th^ edition. Dr. Roth earned his MD and PhD in Biochemistry at the Baylor College of Medicine and completed post-doctoral training at Baylor College of Medicine and the National Institute of Diabetes and Digestive and Kidney Diseases, as well as a residency in anatomic pathology at the National Cancer Institute.

Jason Hassenstab, PhD

hassenstabj@wustl.edu

Dr. Hassenstab is an Assistant Professor of Neurology and of Psychological and Brain Sciences at Washington University, St. Louis. He is the Cognition Core Director at the Dominantly Inherited Alzheimer Network Trials Unit and the Director of Psychometrics for the Charles F. and Joanne Knight Alzheimer’s Disease Research Center, Washington University. His research focuses on the neuropsychology of Alzheimer’s disease and the use of mobile devices to assess cognitive decline in Alzheimer’s disease populations. He has published over 70 articles in peer-reviewed journals. He earned his PhD in Clinical Psychology from Fordham University and completed an internship in clinical neuropsychology at Brown Medical School, followed by further Neuropsychology and Neuroimaging training at Brown Medical School, as well as a Neuropsychology Externship at the Columbia University Medical Center.

### Panel 5: Ethical and Decision-making Skills

Michael Mumford, PhD

mmumford@ou.edu

Dr. Mumford is the George Lynn Cross Distinguished Research Professor of Psychology at the University of Oklahoma. He is a fellow of the American Psychological Association, the Society for Industrial and Organizational Psychology and the American Psychological Society. Previously, he has been a faculty member at the Georgia Institute of Technology and George Mason University, as well as a Research Fellow and Managing Partner for the American Institutes for Research. Over his prodigious career, he has published over 400 peer-reviewed articles and chapters on ethics, leadership, creativity, and planning and has received more than $30 million in research funding. He is on the editorial boards for Leadership Quarterly, Creativity Research Journal, and Journal of Creative Behavior. In addition, he was a recipient of the Society for Industrial and Organizational Psychology’s M. Scott Myers Award for Applied Research in the workplace as well as the Academy of Management’s Eminent Leadership Scholar Award. Dr. Mumford earned his PhD in Industrial Psychology and Measurement from the University of Georgia.

Matthew S. McCoy, PhD

mmcco@pennmedicine.upenn.edu

Dr. McCoy is an Assistant Professor of Medical Ethics and Health Policy at the Perelman School of Medicine, University of Pennsylvania. His research focuses on conflicts of interest in health policy making and ethical issues relevant to medical resource allocation. He will co-teach the course Bioethics and Human Rights in Fall 2018. Dr. McCoy earned his PhD in Political Theory from Princeton University.

Robert Cook-Deegan, MD

bcd@asu.edu

Dr. Cook-Deegan is currently a Professor in the School for the Future of Innovation in Society, Arizona State University. Prior to this position, he was a Research Professor at Duke University for 12 years, where he founded and directed the Center for Genome Ethics, Law & Policy. He has worked in various capacities at the National Academies of Science (1991–2002) and at the congressional Office of Technology Assessment. He is the author of “The Gene Wars: Science, Politics, and the Human Genome,” and has authored over 200 other publications on topics ranging across biomedical research, science and health policies, and intellectual property. Dr. Cook-Deegan earned his MD from the School of Medicine, University of Colorado.

Raymond Tait, PhD

raymond.tait@health.slu.edu

Dr. Tait is the Professor of Psychiatry and Behavioral Neuroscience, previously served as the Vice President for Research at Saint Louis University, a position responsible for the management of intellectual property and university start-ups, among other activities. His research focuses on chronic pain, clinical decision-making, and research ethics. He has published over 100 articles in refereed journals.

Hannah Roth, M.Arch.

hroth22@wustl.edu

Hannah Roth is the Lecturer in architecture at the Sam Fox School of Design & Visual Arts and in the Sustainability Exchange in the environmental studies program , Washington University, St. Louis. She teaches the Materials Research Seminar and Materials Research Seminar: Decoding Sustainability within the architecture program. Concurrently, she sits on the Board of Directors of the US Green Building Council – Midwest Gateway Chapter and is a long-standing member of the Education Committee. Also, she serves on the OneSTL – Water Working Group. In April 2017, she participated as one of five external experts in a workshop for Universal Fibers, an international fiber producer. Setting corporate sustainability goals and framing the first CSR Report was the topic of the Journey + Beyond two-day gathering. Prior to her academic career, she was the Vice President at McCarthy Building Companies Inc. She earned her undergraduate degree and Master of Architecture degree from Washington University, St. Louis.

Amy Waterman, PhD

AWaterman@mednet.ucla.edu

Dr. Waterman is the Professor in Residence at the Division of Nephrology, University of California Los Angeles, and the Director at the Transplant Research and Education Center. She is also the Deputy Director at the Terasaki Research Institute and a Consultant for the UCLA’s Kidney Transplant Program. Her research focuses on kidney transplantation education, as well as identifying and correcting barriers for both donating a kidney and electing to receive a donated kidney. She has contributed to over 100 research articles and book chapters and has been supported by over $22 million in federal grants. As part of her work, she founded the Explore Transplant nonprofit corporation, which helps transplant patients and living donors make informed treatment choices. Dr. Waterman earned her PhD in Social Psychology at Washington University, St. Louis.

Mitch Tyson, MS

mtyson@brandeis.edu

Mitch Tyson is the Principal at Tyson Associates, a Partner in the Clean Energy Venture Group, the Chair and Co-Founder of the North East Clean Energy Council, an Adjunct Professor at the Brandeis International Business School, and the Chair of the Venture CafØ Foundation, and he serves on several corporate, non-profit, and governmental advisory boards. He mentors start-ups through the Cleantech Open, MIT Clean Energy Prize, and MassChallenge competitions; and at Brandeis, he teaches the course, “Building Sustainable Businesses.” Previously, he held the positions of CEO at PRI Automation, a $300M publicly traded semiconductor automation company, the Interim CEO at AmberWave Systems, a VC-backed material science IP company, the CEO at Advanced Electron Beams, a VC-backed clean energy company; prior, he was a Legislative Assistant for Energy and Technology policy to US Senator Paul Tsongas. Mr. Tyson earned a BS in Physics, an MS in Nuclear Engineering and an MS in Political Science from MIT.

Matt Martin, PhD

matthewrmartin@uchicago.edu

Dr. Martin is the Microbiome Lead for the Technology Commercialization Team at the Polsky Center for Entrepreneurship and Innovation, University of Chicago. He has previously worked in several other technology commercialization roles for the University of Chicago and Northwestern University. In addition, he was an Entrepreneur in Residence at Nidus Partners and directed business and technology development at Electrochaea, a renewable fuel company. Dr. Martin earned his PhD in physics from Carnegie Mellon University.

### Panel 6: Competencies

Scott Leisler

SLeisler@dovetail-stl.com

Mr. Leisler is the Co-Founder, President and Chief Creative Officer of Dovetail, a specialized brand communication agency. He has over 20 years of entrepreneurial experience in the creative sphere, having previously co-founded the branding and digital communication company, Big Wheel, as well as the Inferno Media Group. He holds a BFA in Graphic Communications, Graphic Arts, and Art History from the University of Missouri–St. Louis.

Allan Doctor, MD

doctor@wustl.edu

Dr. Doctor is the Professor of Pediatrics and Biochemistry and Molecular Biophysics at the School of Medicine, Washington University, as well as the President and Founding Partner of KaloCyte, Inc. His research focuses on red blood cell signaling in vascular dysfunction during oxidative stress, which has led to over 100 academic publications and several patents. His company is developing a bio-synthetic artificial red blood cell. He earned his medical degree from the University of Virginia and conducted his postgraduate education at the University of Pittsburgh and Boston Children’s Hospital.

Bill Shannon, PhD, MBA

bill@biorankings.com

Dr. Shannon is the Founder and Managing Partner of BioRankings, a data analytics company focused on facilitating and optimizing translational research. He is the former Director of the Biostatistics Consulting Center, Department of Medicine, Washington University, and served for 20 years as a Professor of Biostatistics at Department of Medicine, School of Medicine, Washington University. He has authored and co-authored over 140 peer-reviewed articles and has extensive experience in both solving biomedical data analysis problems himself, as well as leading teams of other experts in consulting projects. He received a PhD in Biostatistics from the University of Pittsburgh and an MBA from the Olin Business School, Washington University, St. Louis. Dr. Shannon is now the Professor Emeritus at the School of Medicine, Washington University.

Pamela Woodard, MD

woodardp@wustl.edu

Dr. Woodard is the Professor of Radiology and Biomedical Engineering at the Mallinckrodt Institute of Radiology, School of Medicine, Washington University. She serves as the Senior Vice Chair and Division Director of the Radiological Research Facilities, the Director at the Center for Clinical Imaging Research, the Head of Advanced Cardiac Imaging, and the Director of the Research Residency Program. She has authored or coauthored over 160 manuscripts, holds several patents and has received many awards for her work. She earned her medical degree from Duke University, and conducted her postgraduate education at the University of North Carolina Hospital at Chapel Hill, the Duke University Medical Center, and the Washington University School of Medicine.

Eric Gulve, PhD

gulve@biogenerator.org

Dr. Gulve serves as the President of BioGenerator, an early-stage evergreen investor in the St. Louis region, and as the Executive Vice President of BioSTL, an organization dedicated to strengthening St. Louis’s bioscience ecosystem and economy through collaborative efforts. Prior to his current positions, he worked for 14 years in the pharmaceutical industry in the field of metabolic diseases and cardiovascular drug discovery research, where he directed laboratories and served on the research leadership teams. In his academic and industry careers, he has directly supervised researchers at educational levels spanning undergraduate to PhD. As such, he is well practiced in mentoring and supervising scientists at different stages in their careers. He earned his PhD in Physiology from Harvard University and conducted his postgraduate work at Washington University studying skeletal muscle glucose transport and metabolism. During his career, he has served as Teaching Assistant, Guest Lecturer, and Course Instructor at the undergraduate, graduate and medical school level.

Rick Hall, PhD

rick.hall@asu.edu

Dr. Hall is a Clinical Professor and the Director at the College of Nursing and Health Innovation, Arizona State University. He has co-founded multiple health-related companies and has taught courses ranging from health technology and communication to leadership and innovation. He is a fellow of the Academy of Nutrition and Dietetics and has spoken at conferences all over the nation on topics of health innovation, entrepreneurship, wellness, and nutrition. He earned his PhD in Hospital Management with an emphasis in Child Nutrition from Iowa State

Vincent Pizziconi, PhD

vincent.pizziconi@asu.edu

Dr. Pizziconi is currently an Associate Professor at the School of Biological and Health Systems Engineering, Arizona State University, and the Founder and Director of the Bioengineering Design and Global Health Technology Center. His research focuses on the development of earth and space bioinspired, biomimetic and bioresponsive materials for multiscale biohybrid diagnostic devices and therapeutic regenerative complex adaptive systems, which has led to over a dozen patents. Over his extensive career, he has been involved in many educational endeavors ranging from K-12 STEM outreach programs to graduate research training and has taught undergraduate and graduate classes related to biomedical product designs such as Biomedical Engineering Capstone Design, as well as “FDA Regulatory Processes and Technical Communications” for medical device product regulation and commercialization, In addition, Dr. Pizziconi has served as a long standing consultant on areas involving both public and private sectors of the medical device and diagnostic industry. This included matters related to the Center for Devices and Radiological Health, the Association for the Advancement of Medical Instrumentation, and the American National Standards Institute, as well as, technical, regulatory affairs, product liability, intellectual property, and related patent infringement issues. He earned his PhD in chemical engineering at Arizona State University.

## Appendix B

**Table B1.** Management panel prioritized learning topics for biomedical I&E (ranked by entrepreneur mean importance)

Topic presented to panelists	Entrepreneur		Intrapreneur	
	Mean importance (5-point scale)	N rating high (4) or essential (5)	Mean importance (5-point scale)	N rating high (4) or essential (5)
**Consensus**				
Knowledge of the different types of funding sources, and how and when to apply for them (venture capital, grants, angel investors, etc.)	4.83	6/6	3.17	3/6
Team management skills: how to lead, coordinate, and trouble-shoot the combined efforts of a group of individuals	4.67	6/6	4.17	5/6
How to create or identify goals?	4.67	6/6	4.33	5/6
Decision-making skills	4.67	6/6	4.5	6/6
Self-management skills: How to take responsibility for your own well-being and behavior; this includes time-management, organization, self-motivation, self-care, and accountability?	4.67	6/6	4.67	6/6
Team-building: How to identify and evaluate the skill sets and capabilities needed for your team?	4.5	6/6	3.83	3/6
Project management skills	4.5	5/6	4.17	5/6
How to define the resources needed for your venture?	4.33	6/6	3.67	4/6
Facilitation skills: How to guide a group through a process in a way that maximizes individual participation and productivity?	4.33	6/6	3.83	4/6
Fundamentals of product adoption: What it takes to get consumers to adopt a new product (work flow, interface, reimbursement strategies, etc.)	4.17	5/6	3.67	3/6
Fundamentals of intellectual property: When and how to file for IP protection, non-disclosure agreements, etc.?	4.17	5/6	3.83	5/6
Fundamentals of product approval: What is involved in getting a drug or device approved?	4.17	5/6	3.83	3/6
How to pitch an idea or otherwise build, improve, and defend business cases?	4.17	4/6	3.67	3/6
How to create a business plan?	4	5/6	3.33	3/6
How to work with outside partners and institutions, including knowledge of what resources are available via contract research and manufacturing organizations and how to manage contracts?	4	4/6	3.67	3/6
No consensus				
Knowledge of regulations	3.83	4/6	3	2/6
Fundamentals of start-up management: How to create a company and administer operations throughout the early stages of development?	3.83	3/6	2.67	1/6
Fundamental business topics: such as finance, accounting, operations, technology, and legal	3.67	3/6	2.67	0/6
How to perform market research: gathering information about consumers, competitors, and current market trends to help inform decisions?	3.67	3/6	3.17	2/6
Interviewing techniques to obtain information from consumers, peers, and competition	3.67	3/6	3.33	2/6
Fundamentals of quality assurance: GLP, GCP, and GMP (good laboratory practices, good clinical practices, and good manufacturing practices)	3.5	3/6	3.17	2/6
Improvisational dialogue skills: how to talk off-the-cuff?	3.33	1/6	3.17	1/6
Familiarity with basic biostatistics tools	3.17	2/6	3.17	2/6

**Table B2.** Vision and imagination panel prioritized learning topics for biomedical I&E (ranked by entrepreneur mean importance)

Topic presented to panelists	Entrepreneur		Intrapreneur	
	Mean importance (5-point scale)	N rating high (4) or essential (5)	Mean importance (5-point scale)	N rating high (4) or essential (5)
**Consensus**				
Communication skills, to engage with diverse types of people and bring different perspectives to the table	4.78	9/9	4.33	7/9
Identifying the assumptions that your business plan relies upon	4.78	9/9	4.22	8/9
Problem formulation strategies, such as how to identify unmet needs in the world	4.67	9/9	4.11	6/9
How to identify your minimum viable product (MVP): the core solution/product behind your ideas to be subjected to potential customer feedback?	4.67	9/9	3.89	6/9
How to perform market research?	4.56	9/9	3.89	7/9
Knowledge of the state of your industry/technology	4.56	8/9	4.22	7/9
How to screen ideas: identify bases for comparison and define criteria against which to evaluate your ideas?	4.44	8/9	4.33	7/9
Understanding what inspires you and motivates you to act	4.44	7/9	3.78	5/9
How to think divergently, and come up with many alternatives given a situation or problem?	4.33	8/9	4.11	8/9
Interviewing techniques to obtain information from consumers, peers, and competition	4.33	8/9	3.89	5/9
Knowledge of what investors want to see before they invest in a venture	4.33	8/9	3.44	4/9
Knowledge of market trends and the underlying models of supply and demand	4.22	8/9	3.44	3/9
Knowledge of entrepreneurial ventures and companies inside your field	4.22	7/9	3.33	3/9
How to find new uses for existing technologies?	4	7/9	3.89	7/9
**No consensus**				
How to assess the intellectual property landscape?	3.78	6/9	3.11	3/9
How to benchmark technologies and identify better ways of doing what’s already on the market?	3.67	5/9	3.56	5/9
Knowledge of entrepreneurial ventures and companies outside of your field	3	2/9	2.44	1/9

**Table B3.** Social skills panel prioritized learning topics for biomedical I&E (ranked by entrepreneur mean importance)

Topic presented to panelists	Entrepreneur		Intrapreneur	
	Mean importance (5-point scale)	N rating high (4) or essential (5)	Mean importance (5-point scale)	N rating high (4) or essential (5)
**Consensus**				
Formal presentation skills, including the use of visual aids	4.67	6/6	4.5	5/6
Active listening skills	4.5	6/6	4.83	6/6
How to effectively give and receive feedback?	4.5	6/6	4.33	6/6
Conflict resolution skills	4.5	6/6	4	4/6
Networking skills: how to establish, maintain, and productively use professional relationships?	4.5	6/6	3.83	3/6
Goal setting strategies	4.5	5/6	4.17	5/6
Fundamentals of team dynamics: how individuals interact in groups, and how to get groups of people to efficiently work together?	4.33	5/6	4.67	6/6
Improvisational dialogue skills/how to talk off-the-cuff	4.33	5/6	4	4/6
Survival skills, such as resilience, managing change, and risk management	4.17	5/6	3.67	3/6
Negotiation skills	4.17	5/6	2.83	1/6
Courtesy skills: being polite, sincere, and sensitive to the needs of others	4.17	4/6	4.33	5/6
How to develop meaningful interpersonal relationships, by caring about others and identifying what matters to them?	4.17	4/6	4.17	4/6
How to build a personal brand: establishing and promoting how you want others to perceive you?	4	6/6	3.33	3/6
How to be assertive: to stand up for your own or others’ rights and needs in a respectful way?	4	5/6	4.33	6/6
Emotion management skills: awareness of your emotions and ability to control your reactions to those emotions in an appropriate manner	4	5/6	4	5/6
How to utilize body language effectively?	4	5/6	3.67	3/6
How to create and deliver elevator speeches?	4	5/6	3.5	3/6
The importance of taking the time to establish norms and expectations in the team	4	5/6	3.33	2/6
How to lead when you don’t have an official position of authority?	3.17	3/6	4.33	6/6
How to “manage up”: establishing and maintaining a productive relationship with your boss?	2.83	1/6	4.33	6/6
**No consensus**				
Metacognition skills: How to be self-aware and self-assess one’s own learning?	3.83	5/6	3.67	4/6
How to cultivate an executive presence/charismatic personality ?	3.83	5/6	3	1/6
Methods for coaching or mentoring others	3.67	5/6	3.5	2/6
Cultivating a growth mindset: having confidence in your skills and in your ability to grow	3.67	4/6	3.5	3/6
Knowledge of different motivational strategies	3.67	3/6	3.5	3/6
Knowledge of different leadership strategies	3.5	4/6	3.5	3/6
How to use Twitter effectively>	2	0/6	1.67	0/6

**Table B4.** Psychological skills panel prioritized learning topics for biomedical I&E (ranked by entrepreneur mean importance)

Topic presented to panelists	Entrepreneur		Intrapreneur	
	Mean importance (5-point scale)	N rating high (4) or essential (5)	Mean importance (5-point scale)	N rating high (4) or essential (5)
**Consensus**				
Identifying your strengths and capabilities, and making the most of them	4.86	7/7	4.71	7/7
How to deal with failure or the threat of failure?	4.71	7/7	4.57	7/7
Methods for measuring progress to inform future decisions, such as “post-mortems” or agile methodologies	4.43	6/7	4.43	6/7
Self-management skills: how to take responsibility for your own well-being and behavior; this includes time-management, organization, self-motivation, self-care, and accountability?	4.43	6/7	4.14	6/7
Networking skills: How to establish, maintain, and productively use professional relationships?	4.29	5/7	4.43	6/7
Using goal-setting as a motivational strategy	4.29	5/7	4.29	5/7
Grit: How to persevere toward goals when it’s tough, and foster a pursuit of passions despite setbacks?	4.29	5/7	4.14	5/7
Knowledge of cognitive biases, such as sunk costs (the unwillingness to leave a project that has failed because one feels that they have put too much effort in it to walk away)	4.29	5/7	4	5/7
Development of interpersonal and team trust to promote candid conversations	4.14	6/7	4.29	6/7
Smart experimentation skills: How to test hypotheses about the environment or about the viability of products or services?	4.14	4/7	4	4/7
How to generate ideas about what could go wrong before a project begins, such as using a “pre-mortem”?	4	6/7	4.14	6/7
How to coach or mentor others?	4	5/7	4	5/7
Self-efficacy: belief in your ability to achieve a goal	4	5/7	3.86	5/7
Person-environment (PE) fit: the idea that matching characteristics, values, and needs between people and their workplaces leads to positive outcomes	4	4/7	3.57	3/7
How to give and receive feedback effectively?	3.86	4/7	4.29	5/7
Emotional intelligence: How to identify and manage your own emotions as well as others?	3.71	5/7	4	5/7
Sensemaking within teams: How to work with a group to make sense of an unexpected event and decide upon a course of action?	3.71	4/7	4.14	5/7
**No consensus**				
Entity (intelligence is unchangeable) vs incremental (intelligence can be increased through effort) theory: the idea that a growth mindset facilitates continued effort after failure rather than helpless responses	3.86	5/7	3.86	5/7
The concept of promotion (pursuing gains) vs prevention (avoiding losses) mindsets when pursuing a goal	3.86	5/7	3.71	4/7
Vicarious learning: using others’ stories to learn in the absence of firsthand experience	3.71	4/7	3.71	4/7
Knowledge of implicit biases: unconscious beliefs about different social groups	3.43	4/7	3.43	4/7
Fundamentals of personality theory: such as big five traits (openness, conscientiousness, extraversion, agreeableness, and neuroticism), individual information processing differences, Myers-Briggs	3.14	3/7	2.86	2/7

**Table B5.** Ethical and decision-making skills panel prioritized learning topics for biomedical I&E (ranked by entrepreneur mean importance)

Topic presented to panelists	Entrepreneur		Intrapreneur	
	Mean importance (5-point scale)	N rating high (4) or essential (5)	Mean importance (5-point scale)	N rating high (4) or essential (5)
**Consensus**				
How to recognize and take stock of your circumstances?	4.75	8/8	4.25	7/8
How to interact ethically with patients?	4.5	8/8	4.62	8/8
Resiliency skills: How to deal with failure, or the threat of failure?	4.5	8/8	3.75	5/8
Ethical interactions with industry and other partner institutions, including potential sources of conflicts of interest	4.5	7/8	4.15	7/8
Ethical pragmatism: keeping your core ethical principles intact, while dealing with difficult practical matters	4.5	7/8	4	6/8
Intellectual property issues	4.5	7/8	3.75	5/8
Questioning your own and others’ judgment: understanding biases, pretenses, and presumptions	4.375	7/8	4.38	7/8
Knowledge of unmet health needs	4.25	8/8	4	7/8
Seeking outside help to make decisions	4.25	7/8	3.88	4/8
Establishing a central vision for what you want and referring back to that vision to guide decisions	4.25	7/8	3.75	6/8
Anticipating consequences of actions: imagining worst-case and most-likely scenarios; asking how actions will affect others; deciding what will always be uncertain	4.25	7/8	3.63	5/8
Implementation considerations: paying attention to operations and being thrifty when expanding in new directions	4.25	7/8	3.38	2/8
Staying up to date on what is happening around the world	4.125	7/8	3.88	6/8
Developing an experimental mindset	4	8/8	4	6/8
How to interact ethically with other professionals?	4	7/8	4.13	8/8
Frameworks for rational economic decision-making, such as Net Present Value calculations	4	7/8	3.25	4/8
Conflicts of interest and the ethical implications underlying them	3.875	5/8	4.13	6/8
Moral reasoning: identifying and weighing the values and interests at stake in different situations	3.875	5/8	4	7/8
Cross-cultural communication	3.875	5/8	4	6/8
**No consensus**				
Using timelines/milestones to evaluate progress for initiatives that have been undertaken	3.875	6/8	3.88	6/8
Issues of power and authority as they affect ethics and risk	3.875	6/8	3.75	5/8
Issues of financial stressors as they affect ethics and risk	3.875	6/8	3.5	5/8
Ethical impacts on different communities	3.875	5/8	3.88	6/8
Knowledge of the Triple Bottom Line (People, Planet, Profit)	3.875	5/8	3.5	4/8
Cultural sensitivity skills	3.875	4/8	3.88	5/8
Emotional intelligence skills: How to identify and manage your own emotions as well as others’?	3.875	4/8	3.75	4/8
Analyzing personal motivations	3.75	6/8	3.5	5/8
Knowledge of the impact that environmental sustainability and climate change have on the global economy	3.625	3/8	3.75	4/8
Career survival skills and considering the long-term impact of your early choices	3.5	3/8	3.63	5/8
Knowledge of health disparities	3.5	3/8	3.38	4/8
The study of ethical and influential leaders	3.375	5/8	3.25	5/8

**Table B6.** Panel 6 responses

Competency	Yes	No	Rationale for no
Planning and management	7	0	
Financial and economic literacy	6	1	Financial planning is a managerial role and not necessarily the role of a basic scientist. This is not needed by everyone and in fact should be a task allocated only to the finance staff of a company, start-up, or university
Mobilizing resources	7	0	
Spotting opportunities	6	1	While this seems like a “yes” answer, in fact basic scientists or managers with a definite task should focus on that and not be looking for “opportunities” wherever they exist.
Vision	5	2	(1) While important to start with a vision, this needs a better definition in entrepreneurship and innovation. A vision may need to be very flexible in this setting, but are sometimes rigid and can be a hinderance for innovation.(2) Again, many people work in a focused area without the need to visualize the future – I want my staff to get the analysis done, and not worry about how or if we will market certain areas for future growth
Valuing ideas	6	1	Value to me is a monetary return and often i do not need staff to worry about this. It is often sufficient for the boss to say do this and not needed for everyone to be able to demonstrate this
Creativity	6	1	Is your future job to plow through a defined scientific problem using established tools, or is it to develop an independent group (lab, start-up, core facility)?
Self-awareness and self-efficacy	7	0	
Mobilizing others	6	1	This is an important leadership trait, but may be delegated to a manager. Effective entrepreneurs are not always the best managers
Working with others	7	0	
Learning through experience	7	0	
Taking the initiative	6	1	Not everyone needs or has leadership skills
Motivation and perseverance	6	1	Not everyone needs or has leadership skills
Coping with uncertainty, ambiguity, and risk	7	0	
Ethical and sustainable thinking	6	1	I don’t believe everyone has the cognitive ability to analyze the consequences of their work
